# Family Dynamics and Elder Financial Exploitation by Family-Member Powers-of-Attorney Agents

**DOI:** 10.1093/geroni/igaa057.1655

**Published:** 2020-12-16

**Authors:** Virginia Vincenti, Bernard Steinman

**Affiliations:** University of Wyoming, Laramie, Wyoming, United States

## Abstract

Elder family financial exploitation (EFFE) is a growing problem likely to increase due to population aging. Older people, often considered vulnerable, are frequently targeted for financial exploitation. Relatives, identified as the largest group of perpetrators often misuse powers of attorney (POA); nevertheless, relationship complexity and dependencies, and family privacy result in underreporting and infrequent prosecutions. The aim of this research is to understand family risk factors that could be used for prevention and early detection. We hypothesized that family dynamics in EFFE families compared to non-EFFE families having family-member POA agents would be at greater risk when these risk factors were present. Our larger multi-state research team created a survey to collect demographic and situational data and to explore family-member and elder characteristics and specific family dynamics that could later place older relatives at risk for EFFE. Analyses consisted of testing whether poor family functioning, ineffective communication/problem-solving dynamics, resource exchange patterns, conflict before/during resource distribution, and entitlement attitudes were statistically associated with the occurrence of EFFE. Specifically, we tested a series of hierarchical logistic regressions to examine the association of family dynamic variables with EFFE. Results suggest that fairness conflict, exchange expectations, entitlement expectations, and communication patterns were statistically associated with EFFE. Current family communication patterns were not a statistical predictor of EFFE. These results could prompt older persons and relevant healthcare, legal, financial, law enforcement, social service, and counseling professionals to work proactively with families and mid-life and older adults to consider risk factors before making end-of-life decisions.

